# Prospecting for Marine Bacteria for Polyhydroxyalkanoate Production on Low-Cost Substrates

**DOI:** 10.3390/bioengineering4030060

**Published:** 2017-06-23

**Authors:** Rodrigo Yoji Uwamori Takahashi, Nathalia Aparecida Santos Castilho, Marcus Adonai Castro da Silva, Maria Cecilia Miotto, André Oliveira de Souza Lima

**Affiliations:** Centro de Ciências Tecnológicas da Terra e do Mar, Universidade do Vale do Itajaí, R. Uruguai 458, Itajaí-SC 88302-202, Brazil; rodrigo.aquicultura@gmail.com (R.Y.U.T.); nathi_zuca@hotmail.com (N.A.S.C.); marcus.silva@univali.br (M.A.C.S.); cecilia.miotto@gmail.com (M.C.M.)

**Keywords:** biopolymer, seawater, waste glycerol, deep sea

## Abstract

Polyhydroxyalkanoates (PHAs) are a class of biopolymers with numerous applications, but the high cost of production has prevented their use. To reduce this cost, there is a prospect for strains with a high PHA production and the ability to grow in low-cost by-products. In this context, the objective of this work was to evaluate marine bacteria capable of producing PHA. Using Nile red, 30 organisms among 155 were identified as PHA producers in the medium containing starch, and 27, 33, 22 and 10 strains were found to be positive in media supplemented with carboxymethyl cellulose, glycerol, glucose and Tween 80, respectively. Among the organisms studied, two isolates, LAMA 677 and LAMA 685, showed strong potential to produce PHA with the use of glycerol as the carbon source, and were selected for further studies. In the experiment used to characterize the growth kinetics, LAMA 677 presented a higher maximum specific growth rate (µmax = 0.087 h^−1^) than LAMA 685 (µmax = 0.049 h^−1^). LAMA 677 also reached a D-3-hydroxybutyrate (P(3HB)) content of 78.63% (dry biomass), which was 3.5 times higher than that of LAMA 685. In the assay of the production of P(3HB) from low-cost substrates (seawater and biodiesel waste glycerol), LAMA 677 reached a polymer content of 31.7%, while LAMA 685 reached 53.6%. Therefore, it is possible to conclude that the selected marine strains have the potential to produce PHA, and seawater and waste glycerol may be alternative substrates for the production of this polymer.

## 1. Introduction

PHAs (polyhydroxyalkanoates) are a class of polyesters produced by prokaryotic microorganisms that are accumulated inside cells as carbon and energy reserves [1,2]. These biopolymers have drawn great interest due to their biodegradability, biocompatibility, the possibility of biosynthesis from renewable resources, and similar physical and chemical characteristics to the main petrochemical polymers [3,4]. Despite the environmental benefits and the potential of using this raw material in several areas, the production costs are relatively high compared to those of conventional polymers [5].

There are bacteria known to be PHA producers, such as *Cupriavidus necator*, *Azohydromonas lata* and *Azotobacter vinelandii*. However, the other organisms capable of using low-cost substrates, accumulating a high PHA content, presenting high productivity, producing copolymers from single carbon sources, and producing other PHA monomer compositions have high economic importance [3,4,6,7]. According to Quillaguamán et al. [8], the marine environment has been poorly explored in terms of prospecting for PHA-producing organisms. However, recent research on halophiles indicates a strong potential for biotechnological production of PHAs, based on a study of the bacterium *Halomonas hydrothermalis*, which was able to accumulate a polyhydroxybutyrate (P(3HB)) content of 75.8% when cultivated in residual glycerol as the only source of carbon [9]. Two other *Halomonas* spp. have been isolated and showed great potential for low-cost PHA production. *Halomonas* sp. TD01 grew rapidly to over 80 g/L cell dry weight (CDW) in a lab fermentor and accumulated a P(3HB) content of over 80% on a glucose–salt medium [10]. *H. campaniensis* LS21 was able to grow in artificial seawater and kitchen-waste-like mixed substrates consisting of cellulose, proteins, fats, fatty acids and starch [11].

There are low-cost ways to synthesize PHAs with the use of halophilic bacteria [12] whose salinity requirements may inhibit the growth of non-halophilic microorganisms, allowing for growth in non-sterile conditions [8,12]. This result was noted in a study by Tan et al. [10], who found evidence of the production of P(3HB) using a non-sterile fermentation process in cultures of *Halomonas* TD01. Moreover, a study by Kawata and Aiba [13] reported the growth of *Halomonas* sp. KM-1 in unsterilized medium cultures. Additionally, seawater can be used as a source of minerals and low-cost nutrients for cultivation, according to a study conducted by Pandian et al. [14] which illustrated the production of PHAs in cultures of *Bacillus megaterium* SRKP-3 (an organism isolated from the marine environment) from seawater, milk residues and rice bran. According to Yin et al. [15], due to their characteristics, using halophiles to produce PHAs can reduce the costs of the fermentation and recovery processes, making them a promising alternative for PHA production.

Therefore, this study describes a search for marine bacteria for PHA production using low-cost substrates, as well as the growth, productivity, and PHA characteristics of the selected producers.

## 2. Materials and Methods

### 2.1. Marine Bacteria

In the present study, marine bacteria maintained in the culture collection of the Laboratory of Applied Microbiology (LAMA) of the University of Vale do Itajaí (UNIVALI) were used. The bacteria were obtained through the South Atlantic MAR-ECO Patterns and Processes of the Ecosystems of The Southern Mid-Atlantic projects. The organisms were isolated from sediment and water samples collected between the surface and a depth of 5500 m from the Mid-South Atlantic ridge, the Rio Grande Rise and the Walvis Ridge.

### 2.2. Qualitative Screening of PHA Producers

All isolates were evaluated based on their ability to synthesize PHAs according to the method described by Spiekermann et al. [16]. Thus, the organisms were cultivated in: (a) Zobell Marine Agar 2216 (AM); (b) AM with added glucose (0.5% w/v); and (c) a mineral medium (MM) with added starch (0.5%), carboxymethyl cellulose (CMC), glycerol, glucose, or Tween 80. The MM composition described by Baumann et al. [17] for 1 L of the medium with a pH of 7.5 was 11.7 g of NaCl, 12.32 g of MgSO_4_·7H_2_O, 0.745 g of KCl, 1.47 g of CaCl_2_·2H_2_O, 6.05 g of Tris (hydroxymethyl) aminomethane (C_4_H_11_NO_3_), 6.65 g of NH_4_Cl, 0.062 g of K_2_HPO_4_·3H_2_O, and 0.026 g of FeSO_4_·7H_2_O. In order to evaluate the potential of the bacteria to produce PHA, all media (except the control) were supplemented with Nile red (final concentration: 0.5 μg/mL), using a stock solution (0.025% m/v in dimethyl sulfoxide). Methods using Nile red are fast and can detect PHA inside intact cells [18,19]; however, the fluorophore can also stain other lipophilic compounds [20]. Organisms were cultivated in an incubator at 28 °C until evident growth occurred (1–9 days). After growth, the colonies were inspected by direct exposure to ultraviolet light (λ = 312 nm; transilluminator UV-TRANS, model UVT-312) and photographed with a digital camera (Canon, EOS Rebel model, Canon Inc., Tokyo, Japan). The fluorescence intensity of isolates is proportional to the PHA content in the cells, as reported by Degelau et al. [21] and Reddy et al. [22]. In this context, the fluorescence intensity was used (Image-Pro Plus, version 6.0, Media Cybernetics Inc., Rockville, MD, USA) as an indicator of polymer content, and the colony area was associated with the growth capacity. The fluorescence intensity and colony area data were statistically analyzed, and the organisms in each test were compared using a Kruskal-Wallis test. In cases of significance (*p*< 0.05), Dunn’s test was applied. Statistical analyses were performed using BioEstat (version 5.3, Instituto Mamirauá; Tefé, Brasil) and Statistica (version 8.0, StatSoft Inc., Oklahoma, OK, USA) software.

### 2.3. Production of PHA in Semi-Solid and Liquid Mediums

The preculture was prepared by inoculating isolated bacterial colonies in Zobell Marine Broth 2216 (CM) or the MM supplemented with glycerol (5%), and cultivating (28 °C; 150 rpm) for 24–48 h. After growth, the preinoculum (10% v/v) was precipitated via centrifugation, and resuspended to assay the PHA production media. Then, the broth was divided into three aliquots (each 100 mL), and cultivated (68 h; 28 °C; 150 rpm; in 250 mL Erlenmeyer flasks) to assay the PHA production. In the first evaluation round, the bacteria were cultured in a MM supplemented with one of the following carbon sources: (a) starch (2% w/v), (b) CMC (2% w/v), (c) glycerol (5% v/v), (d) glucose (5% w/v), or (e) Tween 80 (5% v/v). To evaluate the growth kinetics, cells were cultivated (28 °C; 150 rpm; in triplicate) for 143 h in a MM supplemented with glycerol (5% v/v). Periodically, samples were collected to determine the biomass using gravimetry.

When evaluating the PHA synthesis from the low-cost carbon sources, different media formulations were used (Table 1). For those experiments, residual glycerol from biodiesel production was used, which was provided by a Brazilian company from Rio Grande do Sul (not identified). Selected bacteria were cultivated (28 °C; 150 rpm; 69 h), and the produced biomass was recovered by centrifugation, washed (with distilled water), lyophilized, weighed, and frozen for further analysis by gas chromatography (GC). The PHA quantification analysis was performed using a complex sample that constituted of equal amounts of each replicate. Thus, the PHA content results represent the average of the three cultures.

Assays were also conducted in semi-solid media. Organisms were inoculated (streak) and incubated (28 °C; 6 days) on the MM with 1.8 g·L^−1^ of agar, and separately supplemented with the following carbon sources: starch (2% w/v), CMC (2% w/v), glycerol (5% v/v), glucose (5% v/v) and Tween 80 (5% v/v). After culturing, cells were resuspended in a saline solution, recovered, washed (with distilled water), lyophilized, weighed, and frozen for further analysis by GC (Shimadzu, modelo 17A, Kyoto, Japan).

### 2.4. Quantification of PHA by Gas Chromatography

For PHA quantification, approximately 50 mg of freeze-dried cells were weighed and transferred to a screw-cap tube. Then, to extract the polymer and submit it to the methanolysis reaction to form monomers of methyl esters, 2 mL of H_2_SO_4_/methanol (5:95) was added, along with 2 mL of chloroform and 250 μL of internal standard solution, which was composed of 20 mg of benzoic acid in 1 mL of methanol. Due to the reduced biomass in some assays, 1 mL of chloroform was added during methanolysis. The tubes were then heated (100 °C) in a dry block for 3 h with occasional stirring. After the reaction, the tubes were cooled to room temperature, 1 mL of distilled water was added, and the tubes were vortexed for 30 s and left for phase separation. The chloroform phase was transferred to chromatography vials for analysis. A Shimadzu gas chromatograph (model 17A) equipped with a flame ionization detector (FID) adjusted to 280 °C was used. The temperature program was set to an initial temperature of 50 °C for 2 min, then increased from 50 °C to 110 °C at a rate of 20 °C/min, and finally increased to 250 °C at a rate of 20 °C/min. The injector was maintained at 250 °C, and the oven was maintained at 120 °C. The column used was a VB-WAX (VICI) column, 30 m long, 0.25 mm in diameter, and with 0.25 mm film thickness. The volume injected was 1 μL, with the helium flow set at 1 mL/min, with a total run time of 12 min. The standard curve was made using P(3HB) (Sigma-Aldrich, St. Louis, MO, USA) as an external standard, with a mass ranging from 0.005 to 0.020 g. The standards were submitted to the methanolysis process, as described previously for the bacterial samples.

## 3. Results and Discussion

### 3.1. Screening for PHA-Producing Marine Bacteria

Among the 155 isolates evaluated, 40.6% (63 isolates) presented fluorescence indicative of PHA production in at least one of the growth conditions tested. The results were distributed as follows: 19.4% (30 isolates) were active in one culture condition, 5.8% (9 isolates) were active in two mediums, 5.2% (8 isolates) were active in three mediums, 7.1% (11 isolates) were active in four mediums, 0.6% (1 isolate) was active in five mediums, 1.9% (3 isolates) were active in six mediums, and 0.6% (1 isolate) presented fluorescence in every medium tested (Table 2). Regarding the substrates added in the MM, the number of isolates capable of producing PHA was higher when starch was added, with 30 representatives, followed by 27, 23, 22 and 10 organisms classified as producers in assays supplied with CMC, glycerol, glucose and Tween 80, respectively. In the MM supplied with glycerol, 23 positive isolates (14.7%) were identified as polymer producers, a percentage similar to that observed by Shrivastav et al. [9], who selected PHA producers from the marine environment using residual glycerol as the carbon source, and identified 14% positives using the Nile red method.

When considering the use of agro-industrial by-products as substrates to produce PHA, the great potential of some bacteria can be recognized, particularly the isolates LAMA 674, LAMA 677, LAMA 679 and M 199. The statistical analyses of the colony area and fluorescence on semi-solid media were not significantly different among organisms (data not shown). However, it was possible to identify the organisms with higher fluorescence and growth (area) in each medium, and these were selected for further evaluation. LAMA 679 and LAMA 732 growing in the AM medium were among the highest producers. On the other hand, LAMA 711 and LAMA 644 performed better in the AM medium supplemented with glucose, and LAMA 748 and LAMA 737 performed well in the MM with added starch. In the MM with CMC, LAMA 748 and LAMA 674 performed the best; in the MM with added glycerol, LAMA 685 and LAMA 677 were also sufficient. Finally, when cultivated in the MM with added Tween 80, LAMA 726 and M 199 presented the highest indices.

According to the data provided by the MAR-ECO project, 49.7% of the isolates were obtained from water samples, and 50.3% were obtained from sediments. When considering the sea zonation defined by Hedgpeth [23], 76.6% of the bacteria were collected in the epipelagic zone, 16.9% in the mesopelagic zone, and 6.5% in the bathypelagic zone. In the benthic domain, 29.5% of bacteria were collected from the bathyal zone, and 70.5% were obtained from the abyssal zone. Regarding the sampling locations, 49.7% were from the Rio Grande Rise, 38.7% were from the Walvis Ridge, and 11.6% were from the Mid-South Atlantic ridge. Given this information, the most promising organisms for PHA production were taken from the epipelagic zone, with six organisms, while one isolate was taken from the mesopelagic zone. It is also important to emphasize that 93.5% of the studied bacteria were from the epi- and meso-pelagic zones. To produce PHA, microorganisms need substrates with excess carbon sources, as can be observed in the epi- and meso-pelagic zones [24]. This fact may explain why most of the organisms capable of accumulating PHA were from these zones.

### 3.2. PHA Production in Different Substrates

Among the bacteria screened in the MM assay, those with higher intensities and colony areas were chosen to be evaluated for PHA production in liquid media. As shown in Table 3, production of PHA was not detected in the assays where the media were supplemented with starch, CMC or Tween. However, it is believed that most of these samples were composed of the carbon source itself, so the GC did not detect PHA production. On the other hand, when evaluated in the medium supplemented with glycerol, LAMA 677 had a productivity of 0.0058 g·L^−1^·h^−1^, reaching 1.41 ± 0.18 g·L^−1^ of biomass with 28.28% of a polymer identified by GC as P(3HB). Additionally, LAMA 685 presented better indices with a biomass of 2.03 ± 0.27 g·L^−1^ (32.79% P(3HB)) with 0.0098 g·L^−1^·h^−1^ productivity. A higher P(3HB) production was observed when the media was supplemented with glycerol as a carbon source. This condition was even better than when the media were supplemented with glucose, which is a readily assimilated source. For example, LAMA 685 accumulated 17% more biomass (2.03 g·L^−1^) and 3.4 times more P(3HB) (32.79%) when growing in glycerol compared to glucose as the carbon source. Similar results have been reported previously. Chien et al. [25] evaluated the use of bacteria isolated from mangrove sediments to produce PHA and reported the best PHA content with the use of glycerol in comparison to glucose. According to Chien et al. [25], the organism named M11 produced 30.2% PHA in cells in glycerol culture and 8.1% in the supplied glucose culture. Mahishi et al. [26] used recombinant *Escherichia coli* cultures and reported PHA contents of up to 60% in relation to dry-weight PHA with the glycerol supply, and 38% when supplied with glucose as the carbon source. When evaluating different carbon sources, Mohandas et al. [27] reported that glycerol supported the maximum biomass yield and P(3HB) production, followed by glucose and fructose. The biomass yield and P(3HB) production in the presence of glycerol were 3 g·L^−1^ and 68% (w/w), respectively.

Due to the presence of insoluble substrates in some of the treatments tested, it was difficult to detect PHA production, as the biomass analyzed by GC consisted of both the microbial biomass and the residual substrate. Taking this into consideration, a new set of assays were carried out in a semi-solid medium, as an alternative to obtain cells free from the substrates. For instance, when using media supplemented with CMC, LAMA 674 and LAMA 748 produced PHA as 22.26% and 27.64% of biomass, respectively. Although the substrate used in this study was a source of carbon that is more easily assimilated by microorganisms compared to sources used in other studies, these results are comparable to a study by Van-Thuoc et al. [28]. Van-Thuoc et al. [28] used xylose as the carbon source for PHA production by *Halomonas boliviensis* LC1, and obtained polymer yields ranging from 23.1% to 33.8% of biomass. Bertrand et al. [29] reported a 19.6% accumulation of PHA in cultures of *Pseudomonas pseudoflava* supplied with xylose. Silva et al. [30] reported a PHA content of between 35% and 58.2% when using xylose, and contents of 15.39% to 23.22% when using hydrolyzed sugarcane. Additionally, the PHA accumulation results from this study where Tween 80 was added to the medium were interesting, as isolated LAMA 726 reached a biomass content of 55.24% PHA. In a study by Fernández et al. [31], who used cultures of *Pseudomonas aeruginosa* NCIB 40045, a PHA content of 29.4% was obtained when residual frying oil was added to the culture. On the other hand, He et al. [32] achieved a yield of 63% polymers by cultivating *Pseudomonas stutzeri* 1317 in soybean oil.

### 3.3. Growth Kinetics and P(3HB) Production

As the best productivity was obtained when using glycerol as the carbon source, this condition was further analyzed during bacterial growth. However, the biomass production was much lower than in the previous experiments. For example, LAMA 677 produced 0.16 g·L^−1^ of biomass, of which 22.74% was polymer, compared to the 28.28% of polymer produced in the previous liquid test. This fact can be explained by the longer cultivation time at this stage, allowing for the consumption of the polymer. Moreover, LAMA 685 in the same condition had a 78.63% yield of P(3HB) in the biomass. This value is higher than reported by Cavalheiro et al. [33], who reported a PHA content of 62% when evaluating the production of *Cupriavidus necator* DSM 545 (the main organism used in the industrial production of PHA) under controlled conditions and high cellular concentrations, with a supply of pure glycerol. Mothes et al. [34] cultured the bacteria *Cupriavidus necator* JMP 134 at low cell concentrations using pure glycerol and obtained a 70% accumulation. When compared to other studies, the PHA content of LAMA 685 was similar. Zhu et al. [35] reported a content of 81.9% in cultures of *Burkholderia cepacia* ATCC 17759 grown in a shaker using residual glycerol as the carbonic substrate. Kangsadan et al. [36] cultivated *Cupriavidus necator* ATCC 17699 with pure and residual glycerol and reached contents of 83.23% and 78.26%, respectively. Considering these results, the polymer content obtained in this experiment with LAMA 685 is relevant because it is comparable to that of the organisms used in the production of the polymer on a commercial scale.

Compared to the literature, the maximum specific growth rates obtained in this study were lower, possibly because the medium culture used was poorer in nutrients than those employed in other studies. The values of maximum specific growth rates (μmax) obtained were μmax = 0.087 h^−1^ for LAMA 677 and μmax = 0.049 h^−1^ for LAMA 685. Piccoli et al. [37] studied PHA-producing lines using pure glycerol and found specific speeds ranging from 0.155 to 0.222 h^−1^. Rodrigues [38] reported values of 0.27, 0.24 and 0.23 h^−1^ in glucose, apple cake and starch residues, respectively, in cultures of *Cupriavidus necator* DSM 545. Nascimento et al. [39] cultured *Burkholderia sacchari* LFM 101 on glucose, sucrose and glycerol, but did not observe differences in the maximum specific growth rates obtained with glucose and sucrose (an average of 0.539 h^−1^). However, the highest PHA productivity (0.054 g·L^−1^·h^−1^) was seen with glucose at 35 °C.

### 3.4. Production of P(3HB) in Seawater and Residual Biodiesel Glycerol

An increase in the concentration of pure glycerol in the MM led to a reduction in the dry biomass as well as the PHA content in the cells, as seen in Table 4. When 5% pure glycerol was used in the culture, LAMA 677 produced 1.11 ± 0.04 g·L^−1^ biomass, with 64.28% P(3HB) content, and LAMA 685 produced 0.95 ± 0.06 g·L^−1^ biomass including 43.64% of the biopolymer. When the carbon source increased to 10%, the biomass of LAMA 677 decreased to 0.85 ± 0.05 g·L^−1^, corresponding to a 23.4% reduction in total biomass. In terms of the P(3HB) content, the numbers showed a small decrease when comparing the two concentrations of the carbon source (64.28% at 5% glycerol and 64.04% at 10% glycerol), corresponding to a reduction of 0.24% in the total biomass. The biomass produced by LAMA 685 in the 10% glycerol assay resulted in 0.36 ± 0.07 g·L^−1^, presenting a 62% reduction, and 28.08% P(3HB), presenting a reduction of 15.56%. The negative effect of increasing the concentration of pure glycerol on the growth of organisms has already been reported in other studies. Zhu et al. [35] cultured *Burkholderia cepacia* ATCC 17759 and obtained lower biomass values in the assay of 9% pure glycerol (1.3 g·L^−1^) compared to the assay of 3% of the same carbon source, which yielded 2.8 g·L^−1^.

To evaluate the production of P(3HB) from low-cost minerals and nutrients, a medium composed of seawater and pure glycerol (5%) was used. When comparing this condition to a treatment where the MM was used instead of seawater, a drastic reduction in growth was evident. For instance, the biomass production levels in LAMA 677 and LAMA 685 were approximately 90% and 70% lower, respectively, when using seawater. The seawater used in the medium culture was collected on the Brazilian continental shelf, where the currents have low nutrient concentrations, as reported by Yoneda [40]. These low nutrient concentrations may have led to less growth when compared to the MM. On the other hand, when considering the PHA content in the biomass, the differences were much smaller. For example, LAMA 677 produced approximately 21% less P(3HB) in the seawater cultures. In fact, LAMA 685 exhibited a slight increase when using seawater, reaching 48.26% P(3HB), or a gain of 10%. It is possible that this difference occurred because the collected seawater was poor in nutrients, and this limiting condition favored the accumulation of P(3HB) in LAMA 685.

A low-cost medium composed of seawater as a source of nutrients and minerals, and residual biodiesel glycerol as the carbon source was also tested as a medium for bacterial PHA production. In this case, the medium was supplied with two concentrations of the carbon source, 5% and 10%. The first assay resulted in a biomass and P(3HB) content of 0.06 ± 0.01 g·L^−1^ and 31.7% for the LAMA 677 isolate, and 0.32 ± 0.01 g·L^−1^ and 53.6% for LAMA 685. When using the 10% residual glycerol culture media, LAMA 677 showed no growth, and LAMA 685 presented lower values in relation to the other treatment (5% glycerol). This result was also observed by Zhu et al. [35] in *Burkholderia cepacia* ATCC 17759 cultures with residual glycerol in concentrations ranging from 3% to 9%. The authors reported that the increase in the carbon source concentration resulted in a gradual reduction of biomass and PHA content in the cells. Shrivastav et al. [9] evaluated the growth potential of organisms isolated from terrestrial and marine environments, using residual glycerol at concentrations of 1%, 2%, 5%, and 10% as the carbon sources, and reported high growth in the experiments with 1% and 2% carbon, and reduced growth in the experiments with 5% and 10%. This difference may have occurred as a result of the impurities (salts, esters, and alcohol) in the residual glycerol, which may have affected the metabolic processes of the microorganism. The use of residual glycerol is being investigated as an opportunity to reduce the costs of bacterial PHA production. For example, the new *Pannonibacter phragmitetus* ERC8 isolate was found to be capable of producing PHA (0.43 g·L^−1^) from residual glycerol as the sole carbon source. The maximum PHA production was 1.36 g·L^−1^ when a low concentration (0.80%) of residual glycerol was applied [41]. Naranjo et al. [42] demonstrated the valorization of glycerol for P(3HB) production when working with *Bacillus megaterium*. The study successfully produced 4.8 g·L^−1^ of P(3HB) using 2% (w/v) purified glycerol under controlled conditions. Similarly, Jincy et al. [43] performed statistical optimization for P(3HB) production (0.60 g·L^−1^) using 2% (v/v) residual glycerol by *Bacillus firmus* NII 0830. A study by Hermann-Krauss et al. [6] compared residual glycerol to pure glycerol and did not reveal any negative effects in terms of productivity or polyester properties in *Haloferax mediterranei*. This finding demonstrated that expensive carbon sources for archaic PHA production can be replaced with low residual glycerol phase surplus products from the biodiesel production process.

Conversely, in work carried out by Rodríguez-Contreras et al. [44], the authors obtained high cell dry mass and growth rates when glycerol was used together with glucose in a fermentation with *Cupriavidus necator*. When analyzing the biomass and the growth of *Burkholderia sacchari* using glycerol as a carbon source, the strain properly synthesized P(3HB); however, the biopolymers obtained from both fermentations with glycerol showed low molecular masses.

The impurities present in the residual glycerol vary according to the raw material and the biodiesel production process, as seen from the characterizations. Onwudili and Williams [45] described the composition of a residual glycerol containing 20.8% methanol, 33.1% esters, 1.52% moisture and 2.28% ash. Mothes et al. [34] analyzed residual glycerol from several companies and reported compositions of 5.3% to 14.2% moisture, 0.01% to 1.7% methanol, and 1% to 6% salts. Thompson and He [46] reported extremely variable methanol concentrations, ranging from 23.4% to 37.5%. According to the results obtained in the test with the MM and 5% pure or residual glycerol, the best biomass production was identified with the use of residual glycerol. Under this condition, LAMA 677 reached 1.39 ± 0.05 g·L^−1^, representing an increase of 0.28 g·L^−1^ or 25.2%, compared to the use of pure glycerol. LAMA 685 reached a value of 2.71 ± 0.96 g·L^−1^, with the biomass 2.85 times greater than that recorded in the test with pure glycerol. Thus, the best growth rates occurred when residual glycerol was added to the culture medium. It is possible that this result was obtained as a function of the nutrients contained in the carbon source, as Thompson and He [46] found varying contents of calcium (11.0 to 163.3 mg·L^−1^), potassium (216.7 mg·L^−1^), magnesium (0.4 to 126.7 mg·L^−1^), phosphorus (12.0 to 134.7 mg·L^−1^), sulfur (14.0 to 128.0 mg·L^−1^), and sodium (1.07 to 1.4 mg·L^−1^) when analyzing residual glycerol from several companies.

LAMA 677 had a lower biomass P(3HB) value of 11.34% (absolute value) when grown in residual glycerol (5%) than when grown in a medium with pure glycerol. Other studies have also shown the negative effect of residual glycerol on the accumulation of PHA compared to pure glycerol when used in the same concentrations. This difference also occurred in a study by Kawata and Aiba [13], which used residual and pure glycerol at a concentration of 5% in cultures of *Halomonas* sp. KM-1. The study reported the lowest PHA content in the medium with added biodiesel by-product, compared to purified glycerol. When *Cupriavidus necator* bacteria was cultured in pure and residual glycerol, Posada et al. [47] measured 57.1 g·L^−1^ of PHA in the purified substrate and 27.8 g·L^−1^ in the crude substrate. Kangsadan et al. [36] reported a PHA yield of 83.23% of the biomass with pure glycerol, and 78.26% in the crude form. AndreeBen et al. [48] reported contents of 11.85% of polymer with the use of pure glycerol and 5.24% of PHA with the addition of crude glycerol in cultures of recombinant *Escherichia coli*. This fact can be explained by the influence of impurities in the residual glycerol on PHA synthesis.

In a recent study by de Paula et al. [49], *Pandoraea* sp. MA03 showed strong potential to produce P(3HB) from crude glycerol. Experiments were performed for a 10–50 g·L^−1^ carbon source, and the best values for P(3HB) production were shown in crude glycerol cultivations, compared to pure glycerol, with a polymer accumulation ranging from 49.0% to 63.6% cell dry weight. Based on the P(3HB) production parameters of the evaluated organisms, it is possible to conclude that LAMA 685 has a higher capacity to tolerate impurities in crude glycerol, even though lower growth rates occurred in the medium formulated with seawater and residual glycerol (10%), especially considering that LAMA 677 did not show any growth under the same conditions. Additionally, the cultivation of LAMA 685 in the MM with residual glycerol (5%) resulted in superior biomass, 1.76 g·L^−1^, when compared to the MM with pure glycerol (5%). Moreover, LAMA 677 showed an increase of 0.28 g·L^−1^ in total biomass when using residual glycerol, although the biomass results obtained from both organisms in the MM supplemented with 5% pure glycerol were similar. In addition, LAMA 685 was found to be very similar in P(3HB) content in the assays of the MM with 5% pure or residual glycerol, whereas LAMA 677 achieved a lower P(3HB) content in the residual glycerol cultivation.

The isolates cultured in low-cost media (5% seawater and residual glycerol) expressed a P(3HB) content of 0.02 g·L^−1^ in LAMA 677 and 0.17 g·L^−1^ in LAMA 685. When compared to the results of Pandian et al. [14], who evaluated the PHA production of *Bacillus megaterium* SRKP-3 (isolated organism from the marine environment) in a medium formulated with low-value inputs including seawater, rice bran and dairy residues, the low PHA content is verified, as they obtained results ranging from 0.196 to 6.376 g·L^−1^ of PHA. However, in the study by Pandian et al. [14], the culture medium used for the polymer synthesis could be considered more nutritionally rich, as, according to Silva [50], the effluents of the dairy industry are characterized by high amounts of organic matter, vitamins and minerals. Therefore, the medium culture used by Pandian et al. [14] was composed of higher concentrations of nutrients compared to the medium used in this study, thus possibly explaining the better polymer contents reported.

## 4. Conclusions

It is possible to conclude that marine bacteria have great potential for PHA production. Specifically, the use of marine bacteria from the epipelagic zone, which are exposed to a myriad of substrates, increases the opportunity to accumulate the biopolymer as an energy reserve. This ability was verified in the laboratory when isolates were able to use various carbon sources simulating agro-industrial residues (starch, CMC, glycerol, etc.). Two isolates that were efficient in producing PHA in a high concentration from pure glycerol were further investigated. In addition, the potential of those selected bacteria to synthesize P(3HB) using seawater and residual glycerol from biodiesel as the culture media was revealed. These bacteria will be further characterized in order to optimize their production and evaluate their performance in other low-cost substrates, as well to conduct their molecular identification. These results open a new avenue to explore marine bacteria as efficient converters of by-products into biomass rich in PHA, thus reducing the production costs.

## Figures and Tables

**Table 1:** Culture media formulations used in PHA synthesis assays from different sources of low-cost carbon, minerals and nutrients (residual glycerol and seawater).

Culture Media	Composition
1	90% MM medium * + 5% (v/v) glycerol + 5% distilled water
2	90% MM medium + 10% (v/v) glycerol
3	90% seawater + 5% (v/v) glycerol + 5% distilled water
4	90% seawater + 5% (v/v) residual glycerol + 5% distilled water
5	90% seawater + 10% (v/v) residual glycerol
6	90% MM medium + 5% (v/v) residual glycerol + 5% distilled water

* Marine mineral medium.

**Table 2:** Results of the qualitative analysis of the growth and PHA production of marine bacteria isolates when exposed to two culture media (marine agar and mineral marine) with different formulations.

Isolate LAMA ^3^	MA ^1^		MM ^2^					Isolate LAMA	MA		MM				
	NS	GU	ST	CM	GL	GU	TW		NS	GU	ST	CM	GL	GU	TW
570	−	−	wg	wg	wg	wg	wg	671	−	−	−	+	−	−	−
571	−	−	wg	wg	wg	−	wg	672	−	−	−	−	−	−	−
572	−	−	+	wg	−	−	−	673	−	−	+	+	+	+	−
573	−	−	wg	wg	wg	−	wg	674	+	−	+	+	+	+	+
574	−	−	wg	wg	wg	wg	−	675	−	−	wg	wg	wg	wg	−
575	−	−	wg	wg	wg	wg	wg	677	−	−	+	+	+	−	+
576	−	−	wg	wg	wg	wg	wg	679	+	+	+	+	+	+	+
577	−	−	wg	wg	−	−	wg	680	−	−	−	−	−	−	−
580	−	−	wg	−	−	−	wg	681	−	−	wg	−	−	−	wg
582	−	−	wg	wg	−	−	wg	683	−	−	wg	wg	wg	wg	+
583	−	−	−	wg	−	−	−	684	−	−	wg	wg	wg	wg	−
584	+	−	wg	wg	wg	wg	wg	685	−	−	−	+	+	+	−
585	−	−	wg	wg	−	−	wg	687	−	−	wg	wg	wg	wg	wg
587	−	−	−	−	−	−	−	688	−	−	wg	wg	wg	wg	wg
592	+	−	−	−	−	−	−	689	−	−	−	−	wg	−	−
593	−	−	wg	wg	wg	wg	wg	690	−	−	wg	−	wg	−	−
594	−	−	+	+	+	−	−	691	−	−	wg	wg	wg	wg	−
595	−	−	wg	−	wg	wg	wg	692	−	−	wg	wg	wg	wg	wg
597	−	−	wg	wg	−	wg	wg	693	−	−	−	−	−	−	−
598	−	−	wg	wg	wg	−	−	694	−	−	wg	wg	wg	wg	wg
599	−	−	+	+	−	−	−	695	−	−	−	−	−	−	−
600	−	−	−	−	−	−	−	696	−	−	wg	wg	wg	wg	wg
601	−	−	+	+	−	−	−	697	+	+	−	−	−	+	−
604	−	−	+	−	wg	−	−	698	−	−	wg	wg	wg	wg	wg
606	−	−	wg	wg	wg	wg	wg	699	−	−	wg	wg	wg	−	−
607	−	−	wg	wg	wg	−	−	700	−	−	−	wg	−	−	−
608	−	−	wg	wg	wg	wg	wg	701	−	−	wg	wg	−	wg	wg
610	−	−	wg	−	−	−	wg	702	−	−	+	+	+	+	−
611	−	−	wg	wg	wg	wg	−	703	−	−	+	−	−	−	−
612	−	−	+	+	+	+	−	704	−	−	+	+	−	−	−
613	−	−	wg	wg	wg	wg	wg	705	−	−	−	wg	−	−	−
614	−	−	wg	wg	+	−	wg	706	−	−	wg	wg	wg	wg	+
615	−	−	−	−	−	−	−	707	+	+	−	−	−	−	wg
616	−	−	wg	wg	wg	−	−	708	−	−	wg	−	−	−	wg
617	−	−	wg	wg	wg	wg	−	709	−	−	wg	wg	−	wg	wg
618	−	+	wg	wg	wg	wg	wg	710	−	−	+	wg	−	−	−
619	−	−	wg	wg	wg	−	−	711	+	+	+	+	+	+	−
644	−	+	−	−	−	−	−	712	−	−	wg	wg	wg	wg	−
647	−	−	−	−	−	−	−	713	−	−	wg	wg	wg	wg	wg
650	−	−	wg	−	−	+	−	715	−	−	wg	wg	wg	−	wg
653	+	−	−	−	−	−	+	716	−	−	wg	wg	wg	wg	wg
659	−	−	+	+	+	+	−	717	−	+	−	wg	wg	wg	wg
667	−	−	−	wg	−	−	−	718	−	−	wg	wg	−	−	wg
669	−	−	−	−	−	+	−	719	−	−	+	−	−	−	−
720	−	−	wg	wg	wg	wg	wg	759	−	−	wg	wg	wg	wg	wg
722	−	−	wg	+	−	+	−	760	−	−	+	+	+	−	−
723	−	−	−	−	−	−	−	761	+	+	−	+	+	+	+
725	−	−	−	wg	wg	−	wg	762	−	−	wg	wg	−	−	−
726	+	−	−	wg	−	+	+	763	+	+	wg	wg	wg	−	−
727	−	−	−	wg	wg	−	wg	764	−	−	wg	wg	wg	wg	wg
728	−	−	wg	wg	−	−	wg	765	−	−	+	+	+	+	−
729	−	+	+	+	+	+	−	766	−	−	+	wg	−	−	−
730	−	−	wg	wg	−	−	−	767	−	−	−	−	−	−	−
731	−	−	−	−	−	−	wg	768	−	−	wg	wg	wg	wg	wg
732	+	−	−	wg	wg	−	−	769	−	−	wg	wg	wg	wg	wg
733	−	−	−	−	−	−	−	773	−	−	+	+	+	+	−
734	−	+	−	wg	wg	−	−	775	−	−	wg	wg	−	−	wg
735	−	−	wg	wg	−	+	−	778	−	−	+	−	−	−	−
736	+	−	−	wg	+	+	−	779	−	−	−	−	wg	−	−
737	−	−	+	+	+	+	−	781	−	−	wg	−	−	−	−
738	−	−	wg	wg	−	−	−	782	−	−	−	wg	wg	wg	wg
739	−	−	wg	wg	wg	wg	wg	786	−	−	−	−	−	−	−
741	−	−	wg	wg	wg	−	−	790	−	−	+	−	−	−	−
742	−	−	wg	−	wg	−	−	791	−	−	+	−	−	−	−
743	−	−	wg	wg	−	−	wg	M 112	−	−	wg	wg	wg	wg	wg
744	−	+	wg	wg	wg	−	−	M 135	+	+	wg	wg	wg	wg	wg
746	−	−	wg	wg	−	−	−	M 151	+	−	wg	wg	−	−	−
747	−	−	−	−	−	−	+	M 169	−	−	wg	wg	wg	−	wg
748	−	−	+	+	+	−	−	M 171	+	−	wg	wg	wg	−	−
749	−	−	wg	wg	wg	−	wg	M 173	−	+	wg	wg	−	wg	wg
750	−	−	wg	−	+	+	−	M 180	+	+	+	+	−	−	−
751	−	−	−	−	wg	−	wg	M 189	−	−	−	−	−	−	−
753	−	−	wg	wg	wg	−	wg	M 198	−	−	wg	wg	−	−	wg
754	−	−	+	+	+	−	−	M 199	−	−	+	+	+	−	+
755	−	−	wg	wg	wg	wg	−	M 211	−	−	−	+	−	−	−
756	−	−	wg	wg	wg	−	−	M 84	+	−	wg	wg	wg	wg	wg
757	−	−	wg	−	−	−	wg	M 97	−	−	wg	−	−	wg	wg
758	+	−	−	+	+	+	−								

(+) PHA producer; (−) non-PHA producer; (wg) without growth; ^1^ MA—commercial marine agar medium (NS—not supplemented, and GU—supplemented with glucose); ^2^ MM—mineral medium (ST—supplemented with starch, CM—supplemented with carboxymethylcellulose, GL—supplemented with glycerol, GU—supplemented with glucose, and TW—supplemented with Tween 80). ^3^ Marine bacteria of the Laboratory of Applied Microbiology of the University of Vale do Itajaí.

**Table 3:** Results of the parameters used to analyze the PHA production of the isolates grown in the mineral medium (MM) supplemented with starch, carboxymethylcellulose, glycerol, glucose and Tween 80.

Isolate	Carbon Source	Total Biomass (g·L^−1^)	P(3HB) Concentration (g·L^−1^)	P(3HB) Content in Total Biomass (%)	P(3HB) Productivity (g·L^−1^·h^−1^)
LAMA 748	Starch	16.80 ± 0.97	0	0	0
LAMA 737	Starch	25.31 ± 1.09	0	0	0
LAMA 748	Carboxymethylcellulose	1.19 ± 0.19	0	0	0
LAMA 674	Carboxymethylcellulose	1.24 ± 0.20	0	0	0
LAMA 677	Glycerol	1.41 ± 0.18	0.4	28.28	0.0058
LAMA 685	Glycerol	2.03 ± 0.27	0.67	32.79	0.0098
LAMA 685	Glucose	1.73 ± 0.22	0.17	9.62	0.0025
LAMA 737	Glucose	0.68 ± 0.10	0.05	7.85	0.0008
LAMA 726	Tween 80	0.91 ± 0.08	0	0	0
M 199 A	Tween 80	0.56 ± 0.05	0	0	0

**Table 4:** Results of the parameters used to analyze the P(3HB) production of the isolates LAMA 677 and LAMA 685, in seawater and residual biodiesel glycerol, with different formulations.

Isolate	Culture Medium Composition	Total Biomass (g·L^−1^)	P(3HB) Concentration (g·L^−1^)	P(3HB) Content in Total Biomass (%)	P(3HB) Productivity (g·L^−1^·h^−1^)
LAMA 677	90% mineral medium + 5% glycerol + 5% distilled water	1.11 ± 0.04	0.71	64.28	0.0103
	90% mineral medium + 10% glycerol	0.85 ± 0.05	0.55	64.04	0.0079
	90% seawater + 5% glycerol + 5% distilled water	0.10 ± 0.03	0.03	35.04	0.0005
	90% seawater + 5% residual glycerol + 5% distilled water	0.06 ± 0.01	0.02	31.70	0.0003
	90% seawater + 10% residual glycerol	*	*	*	*
	90% mineral medium + 5% residual glycerol + 5% distilled water	1.39 ± 0.05	0.74	52.94	0.0107
LAMA 685	90% mineral medium + 5% glycerol + 5% distilled water	0.95 ± 0.06	0.42	43.64	0.0060
	90% mineral medium + 10% glycerol	0.36 ± 0.07	0.10	28.08	0.0015
	90% seawater + 5% glycerol + 5% distilled water	0.27 ± 0.03	0.13	48.26	0.0019
	90% seawater + 5% residual glycerol + 5% distilled water	0.32 ± 0.01	0.17	53.60	0.0024
	90% seawater + 10% residual glycerol	0.10 ± 0.02	0.01	10.97	0.0002
	90% mineral medium + 5% residual glycerol + 5% distilled water	2.71 ± 0.96	1.22	44.95	0.0177

* Unobserved growth.
